# Safety profile of 1,008 cases of propofol sedation in the emergency department

**DOI:** 10.1186/cc12330

**Published:** 2013-03-19

**Authors:** S Bradburn, G Lloyd, B Newstead

**Affiliations:** 1 Royal Devon and Exeter Hospital, Exeter, UK

## Introduction

Until recently there were no guidelines for the reporting of adverse events (AEs) during procedural sedation [1,2]. A consensus document released in 2012 by the world SIVA International Sedation Task Force proposed a benchmark for defining AEs [3]. We analysed 1,008 cases of procedural sedation in the emergency department.

## Methods

The study is based on 1,008 patients who received procedural sedation with propofol in the emergency department between December 2006 and March 2012. Patients were selected and sedated to a strict protocol by ED consultant staff. We applied the AE tool by performing a search through patient records, discussion with consultants performing the sedation and consensus opinion.

## Results

From 1,008 cases we identified 11 sentinel (six of hypotension, five cases of hypoxia), 34 moderate, 25 minor and three minimal risk adverse events.

## Conclusion

The study shows a 1% adverse event rate. This supports use of propofol sedation by emergency physicians but within the limits of a strict governance framework. Our safety analysis using the World SIVA adverse events tool provides a reference point for further studies.
